# A thematic analysis of MDMA-related harm and harm reduction experiences and knowledge in Aotearoa New Zealand

**DOI:** 10.1186/s12954-024-01024-8

**Published:** 2024-05-23

**Authors:** Jai Whelan, Ryan D. Ward, Geoff Noller

**Affiliations:** 1 Department of Psychology, University of Otago, William James Building, Level 1, 275 Leith Walk, Ōtepoti/Dunedin, Aotearoa, 9016 New Zealand; 2 Department of General Practice and Rural Health, Dunedin School of Medicine, University of Otago, Ōtepoti/Dunedin, Aotearoa, New Zealand

**Keywords:** MDMA, Reflexive thematic analysis, Aotearoa New Zealand, Harm reduction, Harm

## Abstract

**Background:**

Methylenedioxymethamphetamine (MDMA) is a popular drug worldwide and use is prevalent in Aotearoa New Zealand. Although associated with some significant harms, including fatalities, MDMA is ultimately less harmful than other commonly consumed drugs. We aimed to expand the understanding of MDMA harm and harm reduction strategies from a consumer perspective so that national harm reduction efforts can be better informed.

**Methods:**

We conducted 14 semi-structured focus group discussions including 60 people (aged 18–67, *m*e*dian* = 21) who use MDMA in the Southern region of Aotearoa New Zealand to explore their thoughts and experiences regarding MDMA associated harm and harm reduction. Reflexive thematic analysis was conducted from a critical realist perspective.

**Results:**

Five themes were generated; (1) Mindset and setting matters; (2) Looking after your body and mind, not overdoing it; (3) Other substances increase risk and harm; (4) Trusted friends and peers are protective; and (5) Valid information is key for healthy self-determination; and one subtheme 5.1) Drug checking is essential harm reduction.

**Conclusions:**

We discuss the implications for MDMA consumers and aim to inform national drug policy and the harm reduction practices of consumers and organisations, for the ultimate purpose of reducing MDMA-related harm in Aotearoa New Zealand.

## Introduction

Methylenedioxymethamphetamine (MDMA) is a drug that can produce feelings of euphoria, stimulation, and connection with others via serotonin and other monoamine release [1]. MDMA use is particularly common in club and rave communities [2], as the subjective effects facilitate highly social use and dancing for long periods of time and a sense of connectedness [3, 4]. Some of these effects are also likely to contribute to the drug’s recent success in clinical trials as an adjunct to psychotherapy for post-traumatic stress disorder [5–7].

Although MDMA is rated by experts as less harmful to both the consumer and community than other commonly consumed drugs like cannabis and alcohol [8–11], various harms are associated with consumption, particularly with regular use or high doses [12]. MDMA consumption has also led to deaths across the globe [13], in which hyperthermia, hyponatraemia, dehydration or drug interactions are often contributing factors [14]. Use is also often followed by a “comedown” period, characterised by low mood and fatigue [15]. Additionally, previous research has found evidence of potential neurotoxicity, and mood and memory deficits [16], and although dependence regarding MDMA is controversial, some people report having problems with or controlling use [17, 18]. Beyond MDMA, other novel psychoactive substances sold in place of MDMA (e.g., cathinones) are often associated with significant harm [19, 20]. Importantly, aspects of drug, set and setting [21] such as ecstasy “branding”, personal moods, attitudes and the physical and social environment have also been shown to influence the MDMA experience in negative ways [22, 23].

Drug harm reduction can be conceived as actions that seek to minimise or eradicate the social, health, and economic drug-related harms on individual, community and societal levels [24]. Risks associated with MDMA are often recognised by consumers, and various studies have highlighted behavioural strategies utilized by people who use MDMA to reduce individual risk of harm, including planning use, acquiring from a trusted source, and use of colorimetric reagent testing, among others [25–30]. Drug checking services are also utilised by MDMA consumers, and research regarding these services in Aotearoa New Zealand (hereafter Aotearoa) has found that most people who used a drug-checking service altered their drug-related behaviour and reported increased harm reduction knowledge due to service use [31]. Drug checking was permanently legalised in Aotearoa in 2021, following fluctuations in the national MDMA supply, where synthetic cathinones such as eutylone became prevalent [32]. Despite a relatively high prevalence of 3.6% past year MDMA use in Aotearoa [33], little qualitative research has specifically explored MDMA consumption practices or MDMA-related harm reduction. In a notable exception, Thom [34] found that excessive use and classical comedown experiences were both harmful, but participants reduced this harm through controlled use (moderation) of MDMA, and other substance consumption. Wider consideration of set and setting factors such as overcrowding, high temperatures and improper ventilation, and a lack of friends present, were also described as increasing the likelihood of harm.

Recent changes in MDMA culture and global availability [35] in addition to other drug market and legislative changes within Aotearoa have likely influenced national consumption. Given the clear association with pleasure and positive life impacts reported by MDMA consumers [e.g., 36, 37], understanding current and culturally situated harm reduction thinking and behaviour is critical for safer MDMA consumption. The present research therefore aimed to facilitate a greater understanding of the thoughts and experiences of people who use MDMA in Aotearoa regarding MDMA associated harm and harm reduction.

## Methodology

### Recruitment and participants

Ethical approval for the study was granted by the University of Otago Ethics Committee (ET21/017). The primary researcher, JW (a male in his 20s with MDMA experience, drawn to the research through the electronic music scene of Aotearoa and volunteering with harm reduction organization KnowYourStuffNZ) completed this work as part of his doctorate research requirements. Participants were recruited via word of mouth and advertisements posted around university campus, on community noticeboards (cafes, supermarkets, etc.) and local (Ōtepoti/Dunedin) Facebook™ pages and groups. To meet inclusion criteria, participants had to be aged 18 years or older, have lived in Aotearoa for at least one year, and used MDMA at least once in Aotearoa. A lead (contact) participant was asked to contact other potential participants to participate in the same discussion, so those who participated already knew each other and were already aware of each other’s MDMA consumption. This allowed for more free-flowing and deep discussions between friends without compromising ethical considerations associated with illicit drug research. A $25 NZD supermarket voucher was given to participants to acknowledge their participation.

The total sample consisted of 60 participants across 14 groups in which discussions lasted an average of 117 min. Twelve groups consisted of 4 or 5 participants. Participants ranged in age from 18 to 67 (*M* = 25, *m*e*dian* = 21), 36 of which identified as women and the remainder men (self-reported gender). Most participants identified as belonging to the NZ European/Pākehā ethnic group (78.3%), whilst 5% identified as Māori (indigenous to Aotearoa) or Pasifika, 6.6% identified with an Asian ethnicity, and 15% identified as belonging to another European ethnic group (one individual did not answer). Regarding sexual orientation, 46 were heterosexual (76.67%), 10 were bisexual (16.67%), one was questioning their sexuality (1.67%), and three did not answer (5%).

### Procedure

The study design was based on focus groups, which were semi-structured and conducted in a private campus location or at a participant’s private residence across 2021. JW facilitated all discussions, with a second young male researcher present on seven occasions. Informed consent was audio recorded following agreement to abide by the ground rules of the discussion, which were written by the research team and could be added to by participants. Questions explored participants’ experiences, motivation, harm reduction behaviour, perceptions, and attitudes regarding MDMA use. Most questions were open-ended to maximise depth of discussion, whilst prompts were used to encourage elaboration when necessary. A brief demographic survey was also completed prior to commencement. During discussions, if participants disclosed incorrect or harmful information, the researchers offered the correct information and provided best practice harm reduction advice during or at the conclusion of the discussion to reduce the risk of future harm from MDMA or other drug use. Following discussions, a debriefing sheet that contained various helplines and links to drug harm reduction information was provided and any follow-up questions from participants were answered. Verbatim transcription was completed, including pauses, laughter, and emphasis. All identifying information was removed from transcripts.

### Data analysis

The research was conducted from a critical realist perspective [38], where a participant’s experiences were understood to have basis in reality but are inherently influenced by beliefs that are shaped by a wider cultural context, resulting in ‘situated realities’ [39]. The analysis approach utilised was primarily data-driven, utilising open-coding. This allowed for a broad analysis without attempting to fit data into a particular theoretical context a priori. Reflexive thematic analysis as described by Braun and Clarke [39–41] was conducted and the bulk of analysis was undertaken by JW. In brief, following familiarization with interview transcripts, NVivo (1.7.1; QSR International) software was utilized to generate initial codes that represented features of the data. Coding was both semantic (explicit, surface level) and latent (interpreting underlying meaning or assumption), with no priority given to either type. The first round of coding was fine-grained and conducted within the scope of a broader research project investigating MDMA consumption and culture in Aotearoa. Following this, codes were then revised based on the research aims, collated, and grouped under an initial set of broader themes through an iterative process. These themes were further refined, and underlying codes were checked for fit within each theme. Finally, themes were defined and checked against various data extracts. From the early stages of theme generation until write up, consultation between JW, and GN and RW was carried out to ensure consistency. Quotations below have been edited for brevity and to omit hesitation or repetition unless doing so would alter meaning. Edits for brevity are indicated by “…”. Where words have been edited to aid understanding, this is indicated by “[x]”. Interjections by others are indicated by “|x|”. Pseudonyms used below were assigned by the researchers.

## Findings

The amount of MDMA experience varied across the sample, with use ranging from one to > 100 occasions, with doses generally approximating 100-200 mg per occasion, although this ranged depending on context of use. The average frequency of use ranged from monthly to a few times a year, although some participants reported periods where MDMA was used multiple times a week (e.g., university orientation week), or specific occasions of higher dose consumption (e.g., festivals). Participants’ general understanding of harm reduction also varied significantly, with the concept organically arising early during focus group discussion for many, whilst one participant asked JW “What’s harm reduction?” (Clara). Five coexisting themes were developed through analysis: (1) Mindset and setting matters; (2) Looking after your body and mind, not overdoing it; (3) Other substances increase risk and harm; (4) Trusted friends and peers are protective; and (5) Valid information is key for healthy self-determination. A key subtheme, 5.1) Drug checking is essential harm reduction, was also produced.

### Mindset and setting matters

Mindset was noted as “very important” (Billie) to the reduction of harm before, during and following MDMA experiences. “Getting yourself into the right headspace” (Phoebe) was vital, as being “in a bad mental state” was known to increase the chances of “[having] a bad time” (Tyler). Throughout discussions, participants stressed the importance of mindset in minimizing harm arising from other life stressors or general concern about taking MDMA: “if you’re in like a real bad mood or you’re like, you’re scared about taking it, I feel a lot of the time, you’ll freak out” (Rose). Although most often discussed as a positive amplifier, MDMA could also enhance negative states, as Alex expressed: “[I was in] a bad headspace when I took it, and then that kind of just… like strengthened the anxious feeling that I was having already”. Billie’s account was also telling:

I definitely have to like mentally prepare myself before I take any drugs like, it’s just like a, it couldn’t be a spare of the whim like even after I’ve done it now, someone couldn’t just be like, snort this line, I’d have to like, give myself a good half hour to be like OK… I’m gonna take gear^1^, you know, it’s not like a quick little, oh yeah let’s do it, for me, but that’s just me like, I have to calm my, like talk myself into the situation first without being like, yeah, cause that can turn bad quickly I can imagine… I couldn’t just instantly do it, I’d have to think about it first and just be like, OK this is what’s gonna happen the rest of the night (Billie).

Mindset was not only acknowledged as influencing experience, but a feature that participants actively attempted to control to minimise subsequent harms and was a major factor that could lead one to decide against MDMA consumption. Comedowns were also particularly impacted by mindset, with key affective differences between when “life is so great” and when “everything sucks” (Poppy), with negative mindsets being particularly intensified following intoxication; “…the comedown, if you’re already in a shit space, is fucking hell” (Grace).

Although previous work has highlighted set and setting features as fundamentally distinct [21], mindset was largely described as intertwined with setting, which was also emphasised as critical when trying to reduce harm. Significant feelings of anxiety during the come up could “largely be [attributed] to the environment” (Scarlet). Julie acknowledged that “it’s very setting-dependent”, with various non-party settings resulting in uncomfortable and “odd” experiences. As Liz explained about the tail end of the MDMA high, when “there isn’t any music…you’re getting tired and maybe you’re unable to speak so freely…then I start feeling a little bit paranoid…I really hate that feeling”. Numerous factors are at play (including tiredness), with an “awkward” setting contributing to significant discomfort. These considerations were also to be had when organising “a nice environment to come back to…so that you don’t become depressed or anxious, because you’ve got no need to” (Liz). Furthermore, other people within the setting, and their mindset, could also contribute to harm experiences:

Kate: Compared to the last event that we went to where, it seemed like [security] were literally out to get anyone who looked like they were on something |yeah|.

Steve: It was a bit heavy-handed.

Kate: Which I think made things a little bit more unsafe.

Maria: Yeah I think it stresses people out as well ….

Kate: … cause everyone’s like more on high alert, and so you know, it sort of freaks people out a bit and, you know that’s not the best when someone’s like, necessarily on something, especially if they’re not on something good |yeah|.

The above exchange illustrates the intersection and interaction of “set” and “setting” and the impact this can have on an MDMA experience, where individuals perceived to be unfriendly towards drug use can and do induce stress and feelings of unsafety within consumers of MDMA. Additionally, the presence of relatively neutral others was also explained as a potential contributor to harm:

Keith: …I wouldn’t go and do MD with like, some people I’m not super close with, or like, go out with strangers, cause that just like, I feel like there’d be, too many random thoughts in my head or …too much to worry about, externally, that it would take away from the experience.

…

Fred: …that could be some sort of like you know, mental harm from that aye, if you didn’t minimize enough of the, the worries or whatever aye like you could have a bad experience all together and you know like it’s not necessarily physical harm but you could just have a bad night and, you know, not be good I guess.

The influence of set and setting on MDMA-related harm was not always conceptualised as large or obvious but could arise from deviations in preferred participant settings. In contrast to discussion about harm, an exchange about Anna’s initial MDMA experience was illustrative of the overall impact of mindset and setting:

Maia: … it was really fun and I think because the whole setup was really good as well.

JW: Right. At the event you mean?

Maia: Um, oh just, the whole, yeah the gig plus, the just, the āhua^2^ of the day, evening and, yeah.

Anna: Yeah we had like a, we had like drinks and nibbles with friends at, [Maia’s] house with a beautiful view, and watched fireworks.

…

Anna: …I like cleaned my room perfectly and beautifully before I left, and …I’d gone to the supermarket so that when I came home it would be like, like I knew that I had a safe, like whatever happened, I was gonna be all good.

Here, a positive mindset and well-planned setting, including an organised post-intoxication setting, contributed to and resulted in, a positive āhua^2^ and lack of harm – something which many participants endeavoured to achieve across their MDMA experiences.

### Looking after your body and mind, not overdoing it

Participants were generally aware of physiological impacts of MDMA and endeavoured to reduce physical harms through mindful preloading and postloading with various substances among other protective behaviours. “Eating a good meal before” and “fruit and veges the next day” (Jasmin), having a healthy amount of water, and utilising chewing gum or magnesium to combat jaw clenching were all cited as ways to reduce harm. Supplements such as 5-HTP were also discussed as potentially helpful for “serotonin balance” (Jasmin) whilst others were suggested for neuroprotection and general recovery, with a broad understanding that “putting all the depleted chemicals back in your body” (Liz) was important. Illness was also cited as a reason to reconsider MDMA consumption: “… you need a bit more, get up and go, I think I’d take care of myself and um, get over the bug first, yeah” (Tracy). “Making sure you’re physiologically comfortable” (Liz) through “making sure not to overheat” (Jess), “letting [your] heart chill out for a bit…relax” (Willow), and “[making] sure you’ve got enough warm clothes” (Liz) were also discussed as small but useful ways of ensuring harm was reduced. For some participants, changing the route of MDMA administration was also beneficial:

…I started off with caps and I loved it, and then one time I had a really bad come up, I was like, it just, I felt weird, and then, I only do like lines now…cause then I know how much I’m taking, more obviously, and it hits me way quicker, so I don’t, I don’t like waiting, it makes me nervous (Amanda).

Having greater control over the timing of the experience by changing consumption behaviour was able to quell Amanda’s anxiety, and similar changes to route of administration were able to reduce nausea and vomiting in other cases. Although oral consumption of MDMA is typically the safest route of administration for MDMA [42], for Amanda the effects (or lack of timely effects) associated with oral use were harmful, and more so than harms associated with intranasal use, affirming that harm reduction is often highly contextual and individualised. Additionally, cannabis (and sometimes other substances such as ketamine) was also utilised to “take the edge off” (Eddie), “wind down…chill out” (Grace), and “dilute the comedowns” (John), resulting in a more comfortable MDMA experience overall [25, 27, 28].

Participants were very eager to highlight the importance of taking breaks and not using too much MDMA. All participants perceived MDMA use to have some risk, and most were aware of the involvement of serotonin in the MDMA effect, citing concerns that overuse may lead to “serotonin syndrome”^3^ (Tyler), “depression symptoms” (Steve) and generally “fucking with [your] brain” (Maia). Although high levels of use were occasionally discussed as occurring following initial consumption, changes to dosing and frequency shifted as participants learned to reduce common secondary harms like overstimulation resulting in pain and tightness in the body and sleeping difficulties. Avoiding frequent use was also said to protect against more negative comedown experiences, which were explained as reduced or even absent after break periods, often conceptualised as a sign of body and mind “getting worn out” (David). Another significant concern for participants was “[becoming] dependent on MDMA for a good time” (Amanda). Becoming reliant on MDMA to have fun was a major worry for various reasons:

Because realistically like, you’ve gotta kind of pull yourself away from chasing that high to start off with and kinda have a like mature approach to it because then…like if you have it lots and you get a tolerance you have to have more which is bad for you, which is then more expensive, so then it’s like a, domino effect of it, the more you do like the worse off you’re gonna be…if you still wanna like use it, for [special] occasions…that’s why it’s worth saving it for those kind of things (Luke).

Not only was tolerance, associated elevation in dosing, and subsequent economic harm of concern, but the potential negative implications of continued use and what could be described as psychological dependence. By not consuming in excess and “insuring [oneself] around it” (Grace), participants exemplified “sensible” MDMA use [46] and were able to keep well, maintain other commitments, and reduce risk of negative consequences associated with work and study.

### Other substances increase risk and harm

Direct and indirect narratives regarding harm from other substances sold as MDMA were commonly discussed by participants. While some participants discussed the consumption of amphetamines in place of MDMA, synthetic cathinones such as eutylone, an under researched but relatively potent stimulant implicated in several deaths [47], were a major contributor to harm experiences, causing anxiety and panic attacks, auditory hallucinations, insomnia, tremors, and comedown experiences that were described as longer and more severe than MDMA.

My cousin … has taken eutylone twice now by accident, um, thinking it was MD … some of [his] friends took like, a dose that they would usually take of MD … it was huge for a eutylone dose and they didn’t sleep for like two or three days, they were really paranoid, they said that they were like seeing things, they couldn’t sleep they couldn’t eat, they were just like anxious, paranoid messes. … they were just lucky that they had people there looking after them, and to like, kinda keep them calm, and they knew what was happening they were like, “oh no we’ve got eutylone”, because it had kinda been publicized enough that it’s out there, so, they knew enough about what was happening, but I could really imagine that if you didn’t know what was happening and you didn’t have people there to look after you, you might be like “oh it’s finally happened, I’ve had a psychotic break”, and you might go and do something really dangerous… (Jasmin).

Jasmin’s story about her cousin’s peer group demonstrated not only the direct negative symptoms associated with eutylone consumption, but how harm was facilitated by consuming a dose of the more potent eutylone informed by prior MDMA doses. More general concerns about potential consequences of consumption within an uneducated population were also expressed, where consumers may act in a way that leads to further harm because of the drug effects on one’s mental state. Although cathinones were presumed to cause the most serious harm across participants, other unknown substances also contributed to feeling “sick and anxious” (Ethan) and an overall loss of enjoyment. Experiences of mistaken consumption led to concerns about substance quality, particularly during and after the COVID-19 pandemic, and were “a deciding factor in not doing gear” (Mike).

Other instances included mistaken consumption of substances presumed to be MDMA at the time of consumption but revealed to be another drug shortly thereafter. One participant shared their experience of mistakenly snorting ketamine offered to them at a festival while under the influence of MDMA and alcohol:

… I was just like “oh my god” …and then I, stupidly like started feeling really sick and like, motionless, and I took myself back to the tent and like, … just sat down on the air bed, and then just like all of sudden like, I’m pretty sure I K-holed, because I couldn’t, I was paralysed, and I remember it being like, the most terrifying thing of my life (Rose).

These experiences show that in general, unintended consumption of various other substances led to negative experiences. In some cases, these were enough to lead people to dispose of their drugs. Julie described one instance in which, despite managing to get money back from the friend who sold the other substance: “we all just flushed our stuff down the toilet, we were like … this is not worth the risk”.

Alcohol, which was acknowledged as commonly consumed in conjunction with MDMA, was another drug considered a facilitator of harm. For some, alcohol-related harm was exacerbated by MDMA consumption, which resulted in negative experiences and subsequent behaviour change:

If I haven’t really been drinking or I’ve only had like one or two drinks and then I do some MDMA, I can keep drinking … and I don’t feel drunk, and then like as soon as the MDMA starts wearing off, I realise that I’m actually pretty fucked, and I’m like, don’t wanna get into that territory, so I don’t really drink when I do MDMA, cause I can’t tell how drunk I actually am (Chloe).

Concomitant use also contributed to “worse comedowns” (Chloe) and “more anxiety the next day … hangxiety” (Willow), which did not occur to the same extent in the absence of alcohol consumption. Furthermore, alcohol was also described as reducing feelings of safety through reduced awareness and slowed “reaction time” (Jasmin). Differences in the disinhibiting effects of alcohol and MDMA were underlined by Thomas, who stated that the impacts on “the risk taking is a lot different”, with alcohol facilitating negative behaviours, causing “destruction” (Eddie) and “[doing] dumb shit” (Thomas). Beyond subjective intoxication, physiological risks of concomitant MDMA and alcohol consumption such as “dehydration” (Steve) were also cause for worry. For Jasmin, the combination was of particular concern:

I think that the way that a lot of people take it when they are drinking, is super harmful, just because, I know that they are getting the depressant effect from the alcohol, but then they’re having an upper, and it’s making them feel not as drunk. So then they’ll drink more, and they’ll take more, because they’re cancelling out some of that down, and I do definitely worry when people are doing that, because I’m like, that is definitely a one-way path to something bad happening … going like down that scale of CNS depressant, is gonna end in coma and death… So it’s way more concerning to me when I see that kind of use happening, and I think that’s a really common use cause because people don’t know that that’s a risk (Jasmin).

The increased risk of harm was discussed as not only occurring because of a combined MDMA and alcohol effects, but because alcohol acted as a barrier to harm reduction behaviour, as highlighted by Georgia when she said “…half the time I’m drunk when I take it, and if I guess |*haha*| I’ll take like 3 and I’ll be like why do I feel so high?” Alcohol-induced disinhibition was also explained by Steve as facilitating MDMA-related risk:

… I’ve seen people be like, “I’m not taking MD” or like “I’ll never take MD” and then they’re pissed and they’re like “give me a cap” [yeah]. … I don’t think MDMA is so dangerous but like, that’s a dangerous situation … you don’t know what’s gonna happen to you, essentially? It’s probably not gonna be bad but *haha*, you don’t need to seek out the intoxication on top of what, you already are (Steve).

Albert shared a similar sentiment, “usually … I’m already drunk, when the decision comes, to do gear”. For Leo, alcohol masked thinking about the potential harm of MDMA, reducing cognitive dissonance arising from consumption:

…Psychologically no one wants to know, that they’re doing like drugs that can harm [themselves], so the fact that they’re already like, drinking and it gets them to the stage where they’re like, “oh, I’m drunk enough, I guess, let’s just do gear”, like, that sorta gets rid of the fact that they have to, you know, be sober and realise they’re on potentially [harmful] drugs (Leo).

Although direct MDMA harms may be infrequently realised via consumption, psychological harms may arise from this type of thinking, and continuing to use alcohol in this way is likely to increase risk of harm. Impaired judgement also impacted decision making when being offered a substance:

So I’d like drunk heaps and then went to a random flat, and a guy like offered me … a few like lines … I mean like, I’ve rejected stuff like that before but I was so drunk … so I just like did it, so I can barely remember but, I don’t even know if they told me it was gear I just did a line. And like … just went downhill (Tyler).

Tyler acknowledged that alcohol intoxication was a significant barrier to mindful behaviour, which resulted in a negative experience that would have otherwise been avoided. Mike simplified this point: “If you’re really drunk and someone offers it to you, you don’t really think, it’s like “oh it’s free””. Although alcohol was acknowledged as a significant part of the wider party context, its interplay with MDMA consumption was discussed as enhancing both alcohol and MDMA-related risk. George’s quote “I think, don’t combine it with alcohol aye” exemplified that for some, reduction, cessation, or not initiating alcohol consumption when planning to use MDMA, was worthwhile to mitigate risk.

### Trusted friends and peers are protective

Friends were described by all participants as fundamental to the reduction of harm. The presence of those whom participants trusted was vital to a safer MDMA experience:

I always do it with people I trust, and like … definitely I won’t take it if I’m going out somewhere, if I don’t feel like I have enough of my friends with me to feel like I’m having a safe experience (Jasmin).

During MDMA experiences, participants stated that “it’s good to have sober people around” (Steve) and be open with each other so “everyone knows what everyone’s on” (Kate), so that they may act in the event of an unexpected reaction. Friends were described as key protectors of one another whilst in a “more vulnerable” (Tyler) and “more trusting” (Phoebe) state, also protecting against harm associated with unintended excess consumption. This was even said to extend to comedown experiences, with Harry reasoning that they felt good for the first two days following a 3-day festival because they “had a mean time the night before and [they] were with all [their] mates”. Although post-experience vulnerability can be understood as increased irritability and emotionality [34, 48], perceived personal vulnerability during intoxication was essentially mitigated by the highly social nature of MDMA consumption. Experienced friends were also described as being important for providing assurance to first timers, whilst friends without experience found comfort in sharing their first MDMA experience together.

Sarah: I was quite yeah, unsure of what it would be like, I’ve heard good things, and I was like oh, I’ll give it a go. It was also cause my friends were taking it for the first time as well [yeah], so it was like, let’s do that together [mmm].

Vicki: … for me, it would’ve been harder to take something that, where everyone else has done it before and I’m the only one doing it. Whereas, knowing that there were two others that hadn’t done it, I felt a lot more comfortable in a way.

This comfort extended to consumption of unknown substances, which was highlighted in the following exchange:

Poppy: … I think also, sometimes you don’t really know what’s in it or anything, so I think it, it’s so much safer to do it with, friends |totally|.

Rose: So you can all, like die together *haha*.

Bella: Yeah if you freak out, it’s ride or die |literally|.

Poppy: Yeah literally.

Here, the group joked about the prospect of death via drug poisoning freely, using hyperbole to emphasise the comfort friends provide in the face of uncertainty. Alternatively, an MDMA high could also become anxiogenic in the absence of *trusted* friends:

… I was also not with the people that make me most comfortable, I was with some like kind of, very loose, friends, and it just happened to hit me then. And I remember just, all I wanted to do was find the people I came with, and I couldn’t find them. And so I was freaking out about that (Amanda).

When drug experiences with negative features were described, the presence of friends was commonly discussed as at least partially reducing discomfort, as “you’re kinda like with other people who are in a similar boat, so it’s never like a particularly big deal” (Zoe). Friends and peers were also described as key conduits of information. With many participants explaining that they “got all [their] education from friends… like people that you obviously trust…” (Benji). Intoxication at events was also said to allow one to “engage with other people that are also doing it or know about it” (Will), facilitating information sharing between peers: “someone will know something and be like, ‘did you know this?’” (Will). When considering those in the “rave scene”, sharing information was a form of “[helping] each other out … cause we all want each other to have a good time and a safe time” (Liz), facilitating more positive and less harmful experiences.

Regarding acquisition of MDMA, trusted individuals were also named as key harm reducing agents. Most participants reported primarily buying MDMA from “trusted friends”, or friends of friends with whom a certain level of trust was had. Leo emphasised that although risk is involved in illicit drug consumption, reduction of this risk is a key endeavour: “I can’t say I’ve ever bought off anyone I don’t know, um, when I’m buying I like always buy off a mate. Um, cause, if I’m gonna do drugs, you wanna be able to trust it, right?”. Much like other markets that involve individual-to-individual transactions, trustworthiness is situated as a key virtue that can facilitate smooth interactions and a reduction in negative consequences associated with purchases, as illustrated by this exchange:

Keith: Stranger danger as well like, not getting substance or like MD off people you, don’t know and trust.

George: If you- like if you can’t test it.

Keith: Yeah like if I, cause like I only buy it off people that I trust, and that I would like know that they would do the exact same, like from the same batch or whatever, and like I know that they’re regularly getting things tested, like I’m happy-.

Will: They wouldn’t put you in that situation.

Keith: Yeah … I know like, a lot of the people we buy off are like, socially responsible drug dealers |*haha*|.

Trust is not only outlined as an essential factor for safer MDMA transactions but linked to the wider concept of responsibility. Here, the terms “socially responsible” were used to highlight the importance of those who can be trusted with distribution of quality product, acting as harm reducing agents within the wider MDMA consuming community.

Individuals also expressed the desire to protect others from unidentified substances. David stated, “I wouldn’t be prepared to give it to anybody else unless I’ve tried it and found it was good”, highlighting a level of understanding and trust held between them and their friends that would be broken if harm were to come of providing such a substance, violating normative principles of care within their community. This line of thinking also extended to other circumstances, such as when Poppy discussed an interaction between a friend and their younger sibling who wanted to acquire MDMA:

…she’s come down here and she was like, “can you … get me some like, gear” he was like “oh I don’t know”, blah blah blah, and like he did, you’d obviously rather get it for her and know it’s safe than anything else (Poppy).

The preceding comment exemplifies how harm reduction principles are applied to an MDMA acquisition context, where despite concerns associated with the thought of a younger sibling consuming MDMA for the first time, through the acknowledgment that drug consumption will at some point occur, risk was mitigated through provision of a substance from a trusted source, in this case a sibling. For those who reported purchasing MDMA via online platforms such as Discord or dark net markets, sellers involved in the trade were often discussed as being trustworthy, although such trust was earned via alternative mechanisms to that between real-life relationships.

It’s a good thing about like, the Discord and stuff as well like, they post up the tests and all that kind of stuff, and like you can, get a kind of like, reference system going *haha* like, yeah, so you know you’re not getting eutylone, and methylone and shit like that (Steve).

Trust in those selling MDMA was enhanced via validation of substance quality claims by reviews from others within the community and provided an alternative and valued mechanism by which potential consumers were protected from making a risky purchase that could result in a harmful experience. Tyler also expressed their preference to utilise dark net markets than other, unknown local distributors:

It’s just like safer because it’s more likely to come [from] … further up the distribution [chain] …the vendor’s more like reliable and trusted and stuff. So like, I always try and get from there, over like a random street dealer (Tyler).

Product that was closer to the importer was perceived as less likely to be adulterated than that offered by “street dealers”. Obtaining MDMA via this means was therefore desirable due to greater trustworthiness in said vendors and the lower perceived likelihood that their product would cause harm.

### Valid information is key for healthy self-determination

Information was a major point of emphasis when it came to MDMA-related harm reduction, with access to scientific information and experiential reports via the internet believed to be instrumental for informing ongoing harm reduction efforts at the consumer level. Regarding MDMA information, “…the science behind it and like, the effects, like um, positive, negative, just kind of the overall” (Luna) was considered essential. Information regarding drug interactions, and basic harm reduction information like “how to eat well” (Liz) and “how not to drink too much water” (Tracy) was also underscored. More local information provided by national harm reduction organisations (e.g., The Level and KnowYourStuffNZ) was also thought to be particularly important for Aotearoa-specific harm reduction, exemplified by participants’ attitudes towards the KnowYourStuffNZ pill library, said to be of benefit to some participants who were able to adjust their behaviour when consuming specific MDMA pills. General MDMA-related knowledge held by people both with and without MDMA experience was also thought to be valuable for the purposes of helping others who may be experiencing MDMA-related harm: “Say you go to a concert or something and you see someone who is, having a bad time or something, they can know OK well, this is what we can do” (Will).

However, some participants had concerns about information framing and validity; “I was obviously looking on the internet, umm… about what the consequences were and I saw a lot of stories about, people dying (Megan); “It’s really hard to find good information about that…essentially because there’s not a lot of good sort of, I guess like government information about that stuff, you have to turn to using message boards and stuff like that” (Steve). Although MDMA use was acknowledged to contain some risk, the true nature of this risk as portrayed by some sources was questioned. Within various discussions, knowledge gaps were explicitly identified by participants themselves (“To be completely candid I have no education around what dosage is”, Gabby), and some MDMA-related myths were shared (e.g., colour and form as indicators of quality – “if it’s just rocks you can pretty much tell”, Anna). Many participants expressed a desire to be more informed, and highlighted that a lack of knowledge within people who use MDMA is “pretty scary” (Fred) and allows for greater harm to occur:

Like we don’t even know what we were- you don’t know all the risks and whatever like, the repercussions are… it’s not really like anyone I know or we know is like super educated on it. So, like it’s kind of, yeah, don’t really know (Harry).

Knowledge about MDMA was fundamental to better decision-making and an overall reduction in harm, although many participants thought that information seeking was often catalysed by experiences of harm:

Yeah like, I think everyone needs to know the pros and the cons of anything that they’re taking. And I feel like a lot of people don’t know until it happens to them, like the cons happen to them they’re like “oh crap”, you know? (Julie).

Knowledge was also explained as critical for a greater understanding of the more severe consequences of excessive MDMA use:

I think, people don’t actually sometimes realize the consequences of taking it, like I know a couple [of] people who got, have been like addicted to MD, and had to go to rehab because of it, and I think, especially younger people who are taking more of it now, they don’t- and I know for me when I was taking like a lot of drugs in second year I didn’t really think about the consequences and then, one of my closest friends got diagnosed with like serious psychosis, which was kinda like a, OK, our actions actually have consequences, um, so I think yeah, people not knowing what actually could happen (Willow).

George illustrated the value of information when attempting to make a healthy decision about how one might best use MDMA, or use at all:

… [MDMA is] probably not right for everyone, but like at least, giving them the opportunity, you know, giving a fair chance at making a decision for themselves, cause right now it’s like, they’re told it’s bad, so a lot of people are just gonna leave it there, and there’s probably like gain from…some of those people, changing their perspective. So I don’t know…having them start from a neutral place of like, do I want to or do I not? Or should I, shouldn’t I? And then, also giving them the stuff they need to like, make an informed decision I suppose (George).

Without valid information, participants identified that people who use MDMA, including themselves, are at greater risk of various acute and chronic harms. Although participants stated that most people could acquire and consume MDMA relatively easily, without an adequate level of MDMA-related understanding, consumption decisions were identified as potentially ill-informed. For many, the lack of trustworthy and valid information was explained to minimise the ability for consumers to carry out an accurate and rational cost-benefit analysis to determine what is right for them regarding consumption, or if consumption is right for them at all, within a given context.

#### Drug checking is essential harm reduction

Drug checking was also discussed as a critical part of MDMA-related harm reduction, particularly given that informed behaviour was dependent on confident substance identification, as stated by Fred, “that’s the first step I guess aye…or else the rest doesn’t really matter”. This was said to be important within the current Aotearoa context, where “not being aware of what you’re consuming” (Jack) was the primary concern for participants. Changes in the MDMA supply understood by experienced consumers was thought to put the inexperienced at particular risk: “…young kids out there who are trying it for the first time and they’re not getting something that’s even close to pure, and you know, I think it’s dangerous” (Tracy). “Kids” with limited experiential knowledge tended to also be described as lacking other knowledge that is often afforded to those embedded within the drug culture of Aotearoa for some time.

Participants frequently referred to use of drug checking methods, and although drug checking providers were primarily preferred by those with drug checking experience, personal drug checking with colorimetric reagents was also thought to have some value: “…even if you do it yourself and you buy like a, crappy [reagent] test, just to see if there is anything remotely |legit, yeah|, to what you’re taking…” (Julie). However, these were often thought to lack legitimacy, be considered “pretty unreliable” (Sam) and “not ideal” (Thomas), limited by the nature of the test in the context of the adulterated MDMA supply [see 49, 50], as explained by Jasmin:

Someone was telling me recently that a lot of dealers at the moment have been mixing MD and the cathinones in together so that when people reagent test them it’ll still come up as MD… even though it’s like mostly cathinones, so then they’re like “Oh I tested it, it’s all good”…the only really reliable way to test it is to go and get it spec’d… (Jasmin).

The recently legalised drug checking services were discussed at length, and the spectrometry technology used by these services was generally understood to be better than reagents, carried out by those with relevant and valued drug checking experience: “I trust them more than me” (Anna). The use of these services, particularly KnowYourStuffNZ, was emphasised as not only providing an important service through substance identification, but as a key source of valid harm reduction information. Participants felt that information shared by the service on their website, social media, and directly by volunteers was key for ensuring MDMA consumption was informed and focused on reducing harm, particularly within party contexts: “The sort of information that KnowYourStuff give out at festivals is really good. Advising people on don’t do this with that…that’s great” (Craig).

…the KnowYourStuff people like, probably would definitely have tried it before, or at least had more education surrounding it, because they’re in that field testing and they want to, I suppose, do better. And a lot of them are like, well from what I see, are like young people sorta like us, wanting to make change, and wanting to do better as well (Eddie).

Service volunteers were acknowledged as striving for positive change through harm reduction in a way that was relatable and trustworthy, and their perceived lived experience was seen as a facilitator of that trust. Although some uncertainty existed about certain details of drug checking service provision (availability, process, amount of substance required for checking), participants were overwhelmingly in support:

I think what [KnowYourStuffNZ] are doing is really, really good, and really important. I think it’s making a huge difference. Like I know quite a few people who’ve had it tested and it came back not very good, and you’ve probably saved them from like a lot of, mental harm and trouble because of it, and I think it’s just a really good like initiative to have, you know? Cause at the end of the day like, there’s nothing worse than like, taking drugs which aren’t actually the drugs you think they are, you know? It can be catastrophic, so I think, more funding and more kinda knowledge in that kinda area would be extremely important and beneficial (Georgia).

Although many participants lacked drug checking experience and cited availability issues regarding these services, all participants agreed that drug checking services are essential for providing accurate information regarding substances identity and broader MDMA-related harm reduction. Participants supported greater funding, access and normalisation of drug checking services.

## Discussion

Our analysis resulted in five interrelated themes which broadly highlight how personal harm and harm reduction is conceptualised and experienced within the Aotearoa context: (1) Mindset and setting matters; (2) Looking after your body and mind, not overdoing it; (3) Other substances increase risk and harm; (4) Trusted friends and peers are protective; and (5) Valid information is key for healthy self-determination. Through a deep analysis of broad MDMA-related discussions, we were able to ascertain key understandings of harm and harm reduction and highlight areas of importance for those within this relatively large but under-researched community within Aotearoa.

Unsurprisingly, mindset and setting were discussed as very interrelated and critical for the reduction of harm. Previous research has highlighted negative mindset and unfamiliar or unsafe settings as increasing the likelihood of a negative aspect or experience regarding MDMA [22, 23, 25, 51, 52], which was reflected by many participants. The current findings also reflect much of the previous research concerning knowledge of risks and physical and psychological harms related to MDMA, and behaviours protective against these harm [25–28], including previous reports within Aotearoa [34, 53]. Moderation of MDMA consumption itself was emphasised as critical given discussion of MDMA impacts on the brain [54], with significant concern regarding appropriate use so as not to rely on MDMA for fun [48]. It is positive that many MDMA consumers appear conscious of potential risks and harms associated with high levels of consumption, and other relevant information provided by national harm reduction organizations, although continued communication of associated risks and harm would benefit Aotearoa given the variability of drug education within public schooling [55].

Accidental ingestion of other drugs facilitated harm experiences, particularly eutylone, which aligns with international findings on recent MDMA adulteration and harm [19, 20]. Participant understandings of the risks of other substances compared to MDMA also highlighted a general underlying awareness of the different consequences of uninformed or reckless drug consumption. Unlike the research of McElrath and McEvoy [22], participants in this study generally discussed excessive alcohol consumption unfavourably in the context of MDMA use, citing various interactions with MDMA and the experience that constituted harm. Given that hazardous alcohol consumption is common in Aotearoa [33] and consumed in addition to MDMA, significant consideration of harm reduction interventions relevant to this drug combination should be had, particularly as concomitant use increases the risk of dehydration, hyperthermia and hyponatremia [56]. However, the complexities of drug use often includes both conscious and unconscious acknowledgement of risks and harms, in addition to benefits and pleasures, that are produced within a complex social milieu [57, 58], therefore greater education and understanding of these risks may not result in significant reductions in co-use, particularly given the current place of alcohol within our society and significant pleasures associated with alcohol consumption [59].

Trust of others, including friends and those within the wider MDMA community, was emphasized as particularly important for protecting against harm. This manifested through reducing anxieties, sharing of information, and providing comfort and protection bolstered by highly social use whilst under the influence of MDMA. The significant importance of “community care” [60] for MDMA harm reduction has long been intertwined with MDMA use, particularly within dance party communities and culture [51, 61], and so it is unsurprising that it continues to be a significant part of harm reduction for people who use MDMA in Aotearoa [34]. Because MDMA consumption is a highly social practice, and communities of people who use drugs often share information about best consumption practices [62–64], emphasising the benefits that trusted peers can have for drug harm reduction is likely to facilitate safer consumption practices and promote harm reduction-based norms across the entire community [23]. Assurance about MDMA quality purchased from trusted and known drug sellers [26] or community engagement with darknet markets was also discussed as a key mediator of drug-related harm, which has previously been suggested to exert a positive influence [28, 64–66].

The desire for accessible and valid information was explained as fundamental to healthy decision making within MDMA contexts, although this was often explained as absent or hard to understand, limiting the ability of MDMA consumers to act autonomously with their best interests in mind. Parker and colleagues [67] have suggested that individuals with significant drug knowledge irrespective of experience are “drug-wise”, which can foster healthier consumption practices within communities of people who use drugs. However, such drug wisdom rests upon an understanding that sources of information are trustworthy and valid. MDMA consumer trust in information has been shown to vary by source [68–70], with peers often described as the most accessible, trusted and valuable sources of information [61, 64, 69, 70]. Compared to the early years of MDMA use, information is more available and accessible, particularly via dedicated harm reduction websites and online forums. Although online drug information is largely harm reduction focused [71, 72], trust in the information provided online varies [61, 64], which no doubt mediates the perceived quality and use of this information [73]. The amount of information available means that it is likely that some of the information will be inaccurate or negatively framed, and this volume of information in addition to the diversity of sources may act as a barrier to the accurate appraisal of this information in general [61, 69]. Continued investigation into the validity, perceived trustworthiness, and the sources of information that people who use MDMA utilise will allow for continued resource development and distribution that is useful and trusted by the community, so that harm can be maximally reduced whilst the autonomy of individuals is respected.

Drug checking was also discussed as one of the major ways in which information facilitates healthy self-determination, and the benefits of drug checking have been recognised not only within Aotearoa but in various other jurisdictions [74]. Concerns about the limitations of colorimetric reagents tests for checking MDMA previously expressed in Aotearoa [34] were also shared by our participants. An understanding of the limitations may be more widespread due to the use of spectrometry by drug checking services and increased understanding of these tests in light of more accurate technology and greater harm reduction knowledge sharing. Support for and acknowledgement of the benefits of drug checking services within Aotearoa was clear. However, many participants had not personally utilised the services, often due to lack of accessibility, a major reason also identified in a survey of MDMA consumers in Aotearoa [75]. Although drug checking service provision has continued to expand since legalisation [76], the number of MDMA (or other drug) samples checked across services is still low relative to the estimated population of drug consumers [33]. Thus, further development of public promotion and expansion (e.g., hours of operation, location) of these services is warranted, particularly given the positive impact these services can have on harm reduction knowledge and behaviour within Aotearoa [31, 75]. The lack of quantification technology currently available at client-facing drug checking services [77] also presents as a clear avenue for future development, particularly as high-dose MDMA pills are available globally [35], and in Aotearoa [78].

The knowledge and experience shared by participants may be reflective of the general nature of current day MDMA consumers in Aotearoa, indicating that this population is generally well informed, or “drug-wise” [67], regarding MDMA. This may be in part due to the spread of harm reduction information through communities who use drugs but is also likely a result of greater access to information via the internet, and the development of drug harm reduction in Aotearoa, particularly the legalisation of drug checking and subsequent impacts on drug education and information sharing more broadly. Ultimately, the knowledge exemplified by participants is promising from a public health perspective, reducing concern about uninformed MDMA use that may put consumers at greater risk of harm.

### Limitations

Recruitment of groups of people who knew each other may have skewed our sample towards those who will continue to use, potentially due to less negative experiences, and thus harm experiences may be generally underemphasised within our analysis. However, specific instances of MDMA-related harm and risks were widely acknowledged. The findings may also reflect the experiences of a specific subtype of MDMA consumer or those who use MDMA within the recruitment area, which may be particularly pertinent given that participation was limited to residents of the Southern region of the South Island, who generally use the most MDMA per capita [79]. Given the diversity of people who use MDMA within Aotearoa [53], it is possible that those who have experienced significant MDMA-related harm or those with less MDMA-related harm reduction knowledge may have been underrepresented. Moreover, the sample may represent a specific subset of individuals who are drawn to participate in research due to general personal interest or a desire to increase awareness and decrease harm for other consumers that they see within the wider MDMA culture.

## Conclusion

The nuanced thoughts and experiences of people who use MDMA explored here should be used to inform Aotearoa-specific initiatives that aim to emphasise and promote concepts and actions that MDMA consumers find helpful for reducing harm, whilst also working to minimise risk and sources of harm. Given that drug checking services were highlighted as essential to harm reduction, greater resourcing of service providers is likely to have a major impact on harm resulting from mistaken consumption and increase community knowledge. Within the broader drug landscape and cultural context of Aotearoa, challenges may be faced where alcohol meets MDMA, and alternative and broad interventions and campaigns may be required to reduce these risks. Although Aotearoa is somewhat protected from fast and large shifts in drug markets and culture due to geographical isolation, our unique sociocultural and drug landscape should be considered when developing culturally appropriate and effective harm reduction strategies.

## Data Availability

The datasets generated and/or analysed during the current study are not publicly available for ethical reasons but may be made available from the corresponding author on reasonable request.

## Footnotes

1 “Gear” is often used as a term for MDMA in Aotearoa, particularly by young people.

2 Āhua is a Māori (indigenous people of Aotearoa) word that can be translated to “character” or “nature” – the way that something is.

3 Serotonin syndrome from MDMA alone is rare when compared with use combined with other serotonergic substances or monoamine oxidase inhibitors [43–45].

## References

[1] de la Torre R, Farré M, Roset PN, Pizarro N, Abanades S, Segura M (2004). Human pharmacology of MDMA: pharmacokinetics, metabolism, and Disposition. Ther Drug Monit.

[2] Weir E (2000). Raves: a review of the culture, the drugs and the prevention of harm. Can Med Assoc J.

[3] ter Bogt TFM, Engels RC (2005). Partying hard: party style, motives for and effects of MDMA use at rave parties. Subst Use Misuse.

[4] Sottile JE, Macia KS, Wickham RE, Haug NA (2023). Development and initial validation of an MDMA/Ecstasy motives assessment. Addict Behav.

[5] Heifets BD, Malenka RC (2016). MDMA as a probe and treatment for Social behaviors. Cell.

[6] Mitchell JM, Bogenschutz M, Lilienstein A, Harrison C, Kleiman S, Parker-Guilbert K (2021). MDMA-assisted therapy for severe PTSD: a randomized, double-blind, placebo-controlled phase 3 study. Nat Med.

[7] Mitchell JM, Ot’alora GM, van der Kolk B, Shannon S, Bogenschutz M, Gelfand Y (2023). MDMA-assisted therapy for moderate to severe PTSD: a randomized, placebo-controlled phase 3 trial. Nat Med.

[8] Bonomo Y, Norman A, Biondo S, Bruno R, Daglish M, Dawe S (2019). The Australian drug harms ranking study. J Psychopharmacol.

[9] Crossin R, Cleland L, Wilkins C, Rychert M, Adamson S, Potiki T (2023). The New Zealand drug harms ranking study: a multi-criteria decision analysis. J Psychopharmacol.

[10] Nutt DJ, King LA, Phillips LD (2010). Independent Scientific Committee on D. Drug harms in the UK: a multicriteria decision analysis. Lancet.

[11] van Amsterdam J, Nutt D, Phillips L, van den Brink W (2015). European rating of drug harms. J Psychopharmacol.

[12] Baggott MJ (2002). Preventing problems in Ecstasy users: reduce use to reduce harm. J Psychoactive Drugs.

[13] Roxburgh A, Sam B, Kriikku P, Mounteney J, Castanera A, Dias M (2021). Trends in MDMA-related mortality across four countries. Addiction.

[14] Rigg KK, Sharp A (2018). Deaths related to MDMA (ecstasy/molly): prevalence, root causes, and harm reduction interventions. J Subst Use.

[15] Verheyden SL, Henry JA, Curran HV (2003). Acute, sub-acute and long-term subjective consequences of ‘ecstasy’ (MDMA) consumption in 430 regular users. Hum Psychopharmacology: Clin Experimental.

[16] Parrott AC (2013). Human psychobiology of MDMA or ‘Ecstasy’: an overview of 25 years of empirical research. Hum Psychopharmacology: Clin Experimental.

[17] Degenhardt L, Bruno R, Topp L (2010). Is ecstasy a drug of dependence?. Drug Alcohol Depend.

[18] Uosukainen H, Tacke U, Winstock AR (2015). Self-reported prevalence of dependence of MDMA compared to cocaine, mephedrone and ketamine among a sample of recreational poly-drug users. Int J Drug Policy.

[19] Pascoe MJ, Radley S, Simmons HTD, Measham F (2022). The Cathinone Hydra: increased cathinone and caffeine adulteration in the English MDMA market after Brexit and COVID-19 lockdowns.

[20] Oliver CF, Palamar JJ, Salomone A, Simmons SJ, Philogene-Khalid HL, Stokes-McCloskey N (2019). Synthetic cathinone adulteration of illegal drugs. Psychopharmacology.

[21] Zinberg NE (1984). Drug, set, and setting: the basis for controlled intoxicant use.

[22] McElrath K, McEvoy K (2002). Negative experiences on Ecstasy: the role of drug, set and setting. J Psychoactive Drugs.

[23] Shewan D, Dalgarno P, Reith G (2000). Perceived risk and risk reduction among ecstasy users: the role of drug, set, and setting. Int J Drug Policy.

[24] Newcombe R. The reduction of drug-related harm: a conceptual framework for theory, practice and research. 1992. pp. 1–14.

[25] Allott K, Redman J (2006). Patterns of use and harm reduction practices of ecstasy users in Australia. Drug Alcohol Depend.

[26] Davis AK, Rosenberg H (2017). Specific harm reduction strategies employed by 3,4-methylenedioxymethamphetmine/ ecstasy users in the United States and the United Kingdom.

[27] Kelly BC (2009). Mediating MDMA-related harm: preloading and post-loading among Ecstasy-using youth. J Psychoactive Drugs.

[28] Panagopoulos I, Ricciardelli LA (2005). Harm reduction and decision making among recreational ecstasy users. Int J Drug Policy.

[29] Peacock A, Gibbs D, Price O, Barratt MJ, Ezard N, Sutherland R (2021). Profile and correlates of colorimetric reagent kit use among people who use ecstasy/MDMA and other illegal stimulants in Australia. Int J Drug Policy.

[30] Fernández-Calderón F, Lozano-Rojas Ó, Rojas-Tejada A, Bilbao-Acedos I, Vidal-Giné C, Vergara-Moragues E (2014). Harm reduction behaviors among young polysubstance users at Raves. Substance Abuse.

[31] Hutton F. Drug Checking at New Zealand Festivals [Final Report]. Te Herenga Waka-Victoria University of Wellington; 2020. https://openaccess.wgtn.ac.nz/articles/report/Drug_Checking_at_New_Zealand_Festivals_Final_Report_/13936346

[32] KnowYourStuffNZ. This was the summer of terrible drugs: KnowYourStuffNZ; 2021 [https://knowyourstuff.nz/2021/07/07/this-was-the-summer-of-terrible-drugs/

[33] Annual Data Explorer 2022/23: New Zealand Health Survey [Data File] [Internet]. 2023. https://minhealthnz.shinyapps.io/nz-health-survey-2022-23-annual-data-explorer/

[34] Thom K. ‘Doing Ecstasy in Christchurch’. Ecstasy users’ experiences in relation to drug regulation strategies in New Zealand [Masters Thesis, University of Cantebury]. 2004.

[35] Mounteney J, Griffiths P, Bo A, Cunningham A, Matias J, Pirona A (2018). Nine reasons why ecstasy is not quite what it used to be. Int J Drug Policy.

[36] Elsey JWB, Wuestman VAF, Fieten A. User perceptions of long-term costs and benefits of MDMA use: findings from a large online sample. Drugs: Educ Prev Policy. 2023:1–13.

[37] Hunt G, Evans K (2008). The great unmentionable: exploring the pleasures and benefits of Ecstasy from the perspectives of drug users. Drugs: Educ Prev Policy.

[38] Madill A, Jordan A, Shirley C (2000). Objectivity and reliability in qualitative analysis: Realist, contextualist and radical constructionist epistemologies. Br J Psychol.

[39] Braun V, Clarke V. Thematic analysis: a practical guide. London; Thousand Oaks, California: SAGE; 2022. xxxiv, 338 pages p.

[40] Braun V, Clarke V (2006). Using thematic analysis in psychology. Qualitative Res Psychol.

[41] Braun V, Clarke V (2019). Reflecting on reflexive thematic analysis. Qualitative Res Sport Exerc Health.

[42] Winstock AR. MDMA – how you take it might be more important that you think. Global Drug Survey.2017 [https://www.globaldrugsurvey.com/mdma-how-you-take-it-might-be-more-important-that-you-think/

[43] Makunts T, Jerome L, Abagyan R, de Boer A (2021). Reported cases of serotonin syndrome in MDMA users in FAERS Database. Front Psychiatry.

[44] Parrott A (2002). Recreational Ecstasy/MDMA, the serotonin syndrome, and serotonergic neurotoxicity. Pharmacol Biochem Behav.

[45] Silins E, Copeland J, Dillon P (2007). Qualitative review of Serotonin Syndrome, Ecstasy (MDMA) and the use of other Serotonergic substances: Hierarchy of Risk. Australian New Z J Psychiatry.

[46] Aldridge J, Measham F, Williams L. Illegal leisure revisited: changing patterns of alcohol and drug use in adolescents and young adults. Routledge; 2011.

[47] World Health Organization. WHO Expert Committee on Drug Dependence: forty-fourth report. Geneva: World Health Organization. 2022. https://iris.who.int/bitstream/handle/10665/352462/9789240042834-eng.pdf?sequence=1

[48] Solowij N, Hall W, Lee N (1992). Recreational MDMA use in Sydney: a profile of ‘Ecstasy’ users and their experiences with the drug. Br J Addict.

[49] Murray RA, Doering PL, Boothby LA, Merves ML, McCusker RR, Chronister CW (2003). Putting an Ecstasy Test Kit to the test: harm reduction or harm induction?. Pharmacotherapy.

[50] Winstock AR, Wolff K, Ramsey J (2001). Ecstasy pill testing: harm minimization gone too far?. Addiction.

[51] Hunt GP, Evans K, Kares F (2007). Drug use and meanings of risk and pleasure. J Youth Stud.

[52] Beck J, Rosenbaum M (1994). Pursuit of ecstasy: the MDMA experience.

[53] Hutton F, Australian (2010). New Z J Criminol.

[54] Murphy PN, Wareing M, Fisk JE (2006). Users’ perceptions of the risks and effects of taking ecstasy (MDMA): a questionnaire study. J Psychopharmacol.

[55] Tūturu. What’s happening with AoD education in New Zealand schools? Tūturu. 2023 [https://tuturu.org.nz/stories/whats-happening-with-aod-education-in-new-zealand-schools

[56] van Amsterdam J, Brunt TM, Pierce M, van den Brink W (2021). Hard boiled: Alcohol Use as a risk factor for MDMA-Induced Hyperthermia: a systematic review. Neurotox Res.

[57] Duff C (2008). The pleasure in context. Int J Drug Policy.

[58] Rhodes T (2009). Risk environments and drug harms: a social science for harm reduction approach. Int J Drug Policy.

[59] Wilson N, Gunasekara FI, Thomson G (2011). The benefits and harms of alcohol use in New Zealand: what politicians might consider. N Z Med J.

[60] Span C, Farah B, Ivetìc N, Stronach O. A scoping review of Australian literature on people who use MDMA and their harm reduction practices. Contemporary Drug Problems; 2023.

[61] Duff C, Johnston J, Moore D, Goren N. Dropping, connecting, playing and partying: Exploring the social and cultural contexts of ecstasy and related drug use in Victoria. Melbourne: Premier’s Drug Prevention Council; 2007. https://vgls.sdp.sirsidynix.net.au/client/search/asset/1161131

[62] Becker HS, History (1967). Culture and subjective experience: an exploration of the Social bases of Drug-Induced experiences. J Health Soc Behav.

[63] Van Schipstal I, Mishra S, Berning M, Murray H (2016). Harm reduction from below: on sharing and caring in Drug Use. Contemp Drug Probl.

[64] Jacinto C, Duterte M, Sales P, Murphy S (2008). Maximising the highs and minimising the lows: harm reduction guidance within ecstasy distribution networks. Int J Drug Policy.

[65] Betsos A, Valleriani J, Boyd J, Bardwell G, Kerr T, McNeil R (2021). I couldn’t live with killing one of my friends or anybody: a rapid ethnographic study of drug sellers’ use of drug checking. Int J Drug Policy.

[66] van der Sanden R, Wilkins C, Rychert M, Barratt MJ (2022). Choice’ of social media platform or encrypted messaging app to buy and sell illegal drugs. Int J Drug Policy.

[67] Parker H, Aldridge J, Measham F (1998). Illegal leisure: the normalisation of adolescent.

[68] Gamma A, Jerome L, Liechti ME, Sumnall HR (2005). Is ecstasy perceived to be safe? A critical survey. Drug Alcohol Depend.

[69] Gascoigne M, Dillon P, Copeland J. Sources of ecstasy information: their use and perceived credibility. 2004. Contract No.: Technical Report No. 202. https://ndarc.med.unsw.edu.au/sites/default/files/ndarc/resources/TR.202.pdf

[70] Falck RS, Carlson RG, Wang J, Siegal HA (2004). Sources of information about MDMA (3,4-methylenedioxymethamphetamine): perceived accuracy, importance, and implications for prevention among young adult users. Drug Alcohol Depend.

[71] Soussan C, Kjellgren A (2014). Harm reduction and knowledge exchange—a qualitative analysis of drug-related internet discussion forums. Harm Reduct J.

[72] Barratt MJ, Lenton S, Allen M (2013). Internet content regulation, public drug websites and the growth in hidden internet services. Drugs: Educ Prev Policy.

[73] Kelton K, Fleischmann KR, Wallace WA (2008). Trust in digital information. J Am Soc Inform Sci Technol.

[74] Maghsoudi N, Tanguay J, Scarfone K, Rammohan I, Ziegler C, Werb D et al. The Implementation of Drug Checking Services for People Who Use Drugs: A Systematic Review. Addiction. 2022:532 – 44. 10.1111/add.15734 PMC9299873 34729849

[75] Whelan J, Noller G, Ward RD (2024). Harm reduction behaviours and harm experiences of people who use 3,4-methylenedioxymethamphetamine (MDMA) in Aotearoa New Zealand. Harm Reduct J.

[76] KnowYourStuffNZ. 2 years of legal drug checking today! KnowYourStuffNZ. 2023, November 25 [https://knowyourstuff.nz/2023/11/25/2-years-of-legal-drug-checking-today/

[77] Ministry of Health. Drug checking: Ministry of Health; [https://www.health.govt.nz/our-work/regulation-health-and-disability-system/drug-checking#:~:text=Permanent%20drug%20checking%20licensing%20under,drugs%20for%20drug%20checking%20purposes

[78] KnowYourStuffNZ. Pill Library: KnowYourStuffNZ; [https://knowyourstuff.nz/pill-library/

[79] New Zealand Police. Wastewater Drug Testing in New Zealand: National Overview. Quarter Four: October - December 2021. New Zealand Police. 2022. https://www.police.govt.nz/sites/default/files/publications/wastewater-results-quarter-4-2021.pdf

